# Plasticity is a locally adapted trait with consequences for ecological dynamics in novel environments

**DOI:** 10.1002/ece3.7813

**Published:** 2021-07-21

**Authors:** Matthew N. Bond, Stuart B. Piertney, Tim G. Benton, Tom C. Cameron

**Affiliations:** ^1^ School of Life Sciences University of Essex Colchester UK; ^2^ The School of Biological Sciences University of Aberdeen Aberdeen UK; ^3^ Faculty of Biological Sciences University of Leeds Leeds UK

**Keywords:** age‐at‐maturity, competition, density dependence, eco‐evolutionary dynamics, evolution, life history traits, phenotypic plasticity, size‐at‐maturity

## Abstract

Phenotypic plasticity is predicted to evolve in more variable environments, conferring an advantage on individual lifetime fitness. It is less clear what the potential consequences of that plasticity will have on ecological population dynamics. Here, we use an invertebrate model system to examine the effects of environmental variation (resource availability) on the evolution of phenotypic plasticity in two life history traits—age and size at maturation—in long‐running, experimental density‐dependent environments. Specifically, we then explore the feedback from evolution of life history plasticity to subsequent ecological dynamics in novel conditions. Plasticity in both traits initially declined in all microcosm environments, but then evolved increased plasticity for age‐at‐maturation, significantly so in more environmentally variable environments. We also demonstrate how plasticity affects ecological dynamics by creating founder populations of different plastic phenotypes into new microcosms that had either familiar or novel environments. Populations originating from periodically variable environments *that had evolved greatest plasticity* had lowest variability in population size when introduced to novel environments than those from constant or random environments. This suggests that while plasticity may be costly it can confer benefits by reducing the likelihood that offspring will experience low survival through competitive bottlenecks in variable environments. In this study, we demonstrate how plasticity evolves in response to environmental variation and can alter population dynamics—demonstrating an eco‐evolutionary feedback loop in a complex animal moderated by plasticity in growth.

## Graphical abstract

Phenotypic plasticity is expected to evolve in variable environments, but the consequences this might have for population dynamics in a changing world are currently unknown. Here, we show that plasticity in life history traits can evolve which in turn modulates population dynamics in novel conditions. This evidences the potential role of plasticity in moderating eco‐evolutionary feedback loops.

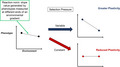

## 1 INTRODUCTION

Phenotypic plasticity (hereafter plasticity) is the capacity of a given genotype to express different phenotypes according to the environments they experience (Fusco & Minelli, 2010; Price et al., 2003). Plasticity, therefore, may facilitate organisms to survive across a range of environmental conditions. (DeWitt et al., 1998; Murren et al., 2015). In the context of a rapidly changing world, plasticity is a key mechanism by which populations might be able to persist in the future (Fox et al., 2019).

**FIGURE 1:**
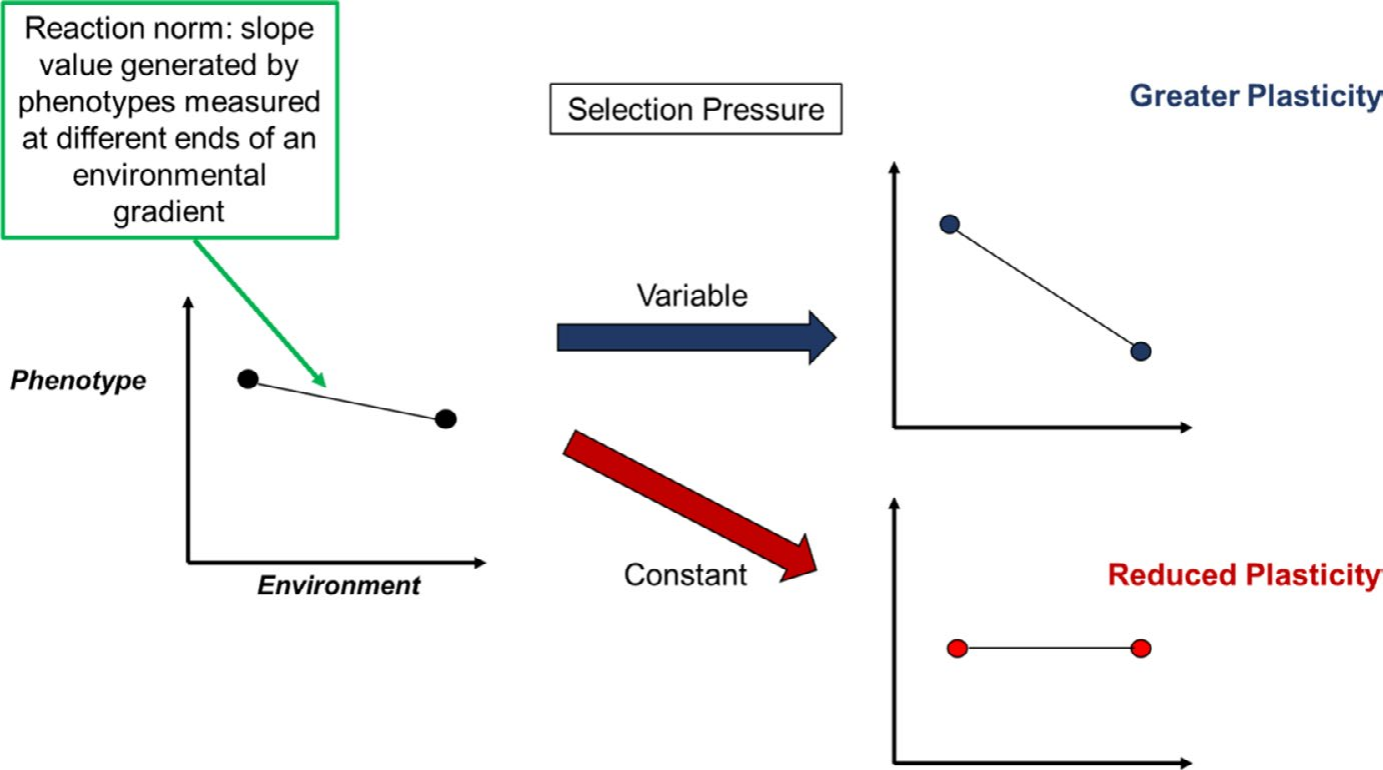
Theoretical predictions for the evolution of plasticity, and therefore change in reaction norm, under constant or variable periods of selection pressure

It is well reported that environmental variability should select for plasticity (Chevin & Lande, 2015). This is based on the premise that the ability to be plastic for a given trait may improve fitness in the face of environmental change (Fox et al., 2019; Gratani, 2014). Both theoretical (Murren et al., 2015) and empirical work (Furness et al., 2015) highlight the importance of the predictability of environmental cues for promoting the evolution of plasticity. Therefore, we might expect that a population exposed to variable selection pressures in variable environments would result in greater plasticity, versus a population exposed to a constant environmental selection pressure where plasticity may erode; especially, if it is costly to maintain (DeWitt et al., 1998; Sereda et al., 2014; see Figure 1 for details). The role of unpredictable stochastic environments in selecting for or against plasticity in populations is less clear. Random environments are predicted to increase the likelihood of plasticity, but possibly nonadaptive plasticity which could promote phenotypic mismatch (Ashander et al., 2016; Reed et al., 2010). Phenotypic mismatch may result in more plastic genotypes being selected against—and again plasticity eroding or being stationary (Ashander et al., 2016; Leung et al., 2020; Oostra et al., 2018). Theoretical predictions for the evolution of plasticity state that predictable variation in selection pressure will result in greatest prevalence of plastic genotypes (Chevin & Lande, 2015). Given that life history evolution allows for improved fitness against conspecifics (Allen et al., 2008; Bassar et al., 2016; Wilson, 2014), we might expect the ability to be flexible (sic plastic) in your life history should evolve in response to predictable but fluctuating intensity of competition. So we can predict that plasticity should evolve in predictably variable environments, erode in constant environments, and erode or be stationary in randomly variable environments (Chevin & Lande, 2015; Leung et al., 2020; Manenti et al., 2015).

Environmental selection on life history traits has been shown to result in “Eco‐evolutionary dynamics”, where reciprocal interactions or “feedback loops” occur between changes in mean trait values and ecological dynamics (Brunner et al., 2019; Cameron et al., 2013, 2014). So for example, intraspecific competition for resources is known to select for shifts in life history and therefore mediate eco‐evolutionary dynamics (Bashey, 2008; Hendry, 2016; Plaistow & Benton, 2009; Schrader & Travis, 2012), it remains unclear how plasticity in those same traits might do the same.

Plasticity in traits associated with life histories, such as reproductive output, body size, and somatic growth, has been linked not only to individual fitness (Acasuso‐Rivero et al., 2019) but also to population level persistence and dynamics (Ashander et al., 2016; Chevin & Lande, 2011; Fischer et al., 2014). The magnitude of environmental variation affects the prevalence of genotypes that express plasticity in reproductive and growth‐related traits, such as in calanoid copepods (Ortega‐Mayagoitia et al., 2018; Sereda et al., 2014) or *Drosophila* sp. (Manenti et al., 2015). While we can measure or estimate plasticity either through time or after exposure to certain environmental conditions to determine how ecology has influenced plasticity, it remains unclear what the feedback of any evolved plasticity has on ecological dynamics experienced by individuals in a population.

**FIGURE 2:**
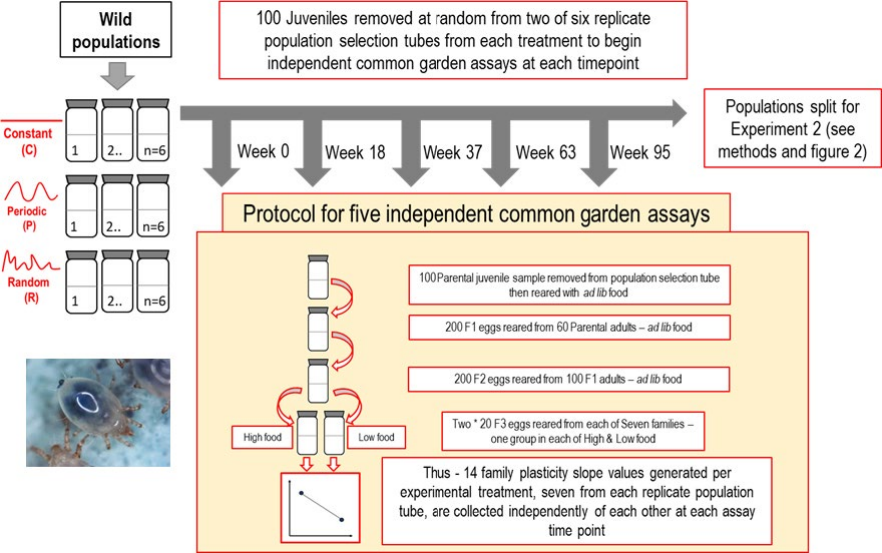
Outline of experimental setup for Experiment 1. All common gardens followed a standard protocol shown here, outlined in the methods and in Cameron et al. (2013), generating measurements of plasticity for each selection tube at five separate time points. Parental mites from two replicate population tubes, of six, per treatment were used to start the common garden assay to estimate life history characteristics—plasticity in age and size at maturity

Here, we investigate the role of variation in resource availability (and therefore density‐dependent competition as a proxy for environmental variation), on evolution of plasticity in life history traits using an invertebrate model system, the soil mite *Sancassania berlesei*. *S*. *berlesei* are a proven model system for studying cohort dynamics, plasticity, and the consequences of the evolution of life history traits in density‐dependent conditions (Benton & Beckerman, 2005; Benton et al., 2001; Plaistow & Benton, 2009). Previous experiments with this system have shown that wild mites when introduced to microcosms experience strong density‐dependent competition, resulting in an initial “extinction trajectory”, before adaptation occurs to delay mean growth to maturity under the novel high‐competition environments (Cameron et al., 2013, 2014). Here, we exploit this experiment and undertake a new analysis investigating the temporal dynamics of evolved plasticity of those same developmental traits, measured in repeated common garden (CG) life history assays where small cohorts are raised in more or less competitive conditions (Figure 2).

We then use a new experiment to investigate how plasticity in life history traits that emerges from the long‐term experiments feedback to determine dynamics of mite population abundance when they are introduced to novel environments. We therefore investigate the role of evolved plasticity in mediating future ecological population dynamics, which via changes in demography, competition, and selection pressures could create an eco‐evolutionary loop.

## 2 METHODS

### 2.1 Study species

Soil mites (*S. berlesei*) are a well‐studied model system in population and evolutionary ecology. They exhibit several instars, before reaching maturity which can vary between 4 and 50 days after hatching (Benton & Beckerman, 2005). There are many factors that affect juvenile growth and survival, where current or ancestral conditions are known to alter mean growth trajectories (Plaistow et al., 2007). Current conditions include an individual’s access to resources including food supply which varies with population density and competitive success (which may depend on size and age). Furthermore, a female may be reproductively active for days to weeks. The end result is a mixed population with overlapping generations. As such, estimations of generation time within selection experiments are presented as a range as opposed to an exact value.

### 2.2 Experiment 1: Evolved changes in plasticity

Wild mites were collected from four UK locations, mixed, and reared with ad libitum food for ca. 2 generations. Mites were then transferred to population tubes with a standardized inoculum of 300 adults (50:50 male to female ratio) and 1,000 juveniles. Population tubes consisted of 25 mm diameter × 50 mm tall glass population tubes half filled with standardized calcium sulfate substratum. Each population tube was subject to an environmental variation treatment: Constant, periodic, or random food supply, with six replicate tubes per treatment level.

Populations were fed with dried balls of activated yeast and two drops of distilled water per day to maintain humidity levels. All populations received the same mean food over a 28‐day period—a mean rate of two 0.0015 g balls of yeast per day. The rate at which food was supplied is defined as the environmental treatment.

The periodic treatment followed a repeating pattern: 9 days 0 balls, 3 days 1 ball, 2 days 3 balls, 9 days 4 balls, 3 days 3 balls, and 2 days 1 ball. This created an environment with predictable variation in cues associated with resource availability.

The random treatment used a random number generation of between 0 and 12 balls yeast per day over 56 days, constrained to no more than 112 balls in that period. This represents an unpredictable variable environment.

Lastly, the constant food supply consisted of two 0.0015 g balls per day; this represents a less variable and predictable environment. Further information on this experimental design can be found elsewhere as data from this experiment has been used to test other hypotheses (Cameron et al., 2013, 2016). This experiment lasted 95 weeks (~13–30 generations, see comments above on study species).

### 2.3 Assessing evolution of plasticity: Life history assay

A time series of CG assays were designed to assess evolved changes in plasticity of life history traits. These assays consisted of five multi‐generation CG experiments that were started by an independent sample (or “cohort”) taken from experimental populations at different points in time. The CG minimizes maternal environment effects as shown in previous studies with this species (Plaistow et al., 2006). The time series of assays allowed for estimation of evolved changes in plasticity uninfluenced by conditions within each population selection tube at any given time, that is, parental and cohort effects (Beckerman et al., 2003; Plaistow & Benton, 2009). Assays were conducted on weeks 0 (initial wild‐type assay), 18, 37, 63, and 95 (Figure 2).

The assay begins by random removal of juveniles from two replicate experimental population tubes for each environmental treatment (Parentals *n *= c100; Cameron et al., 2013). These parental generation juveniles are reared with *ad lib* food, and 30 male and 30 females are mated and allowed to lay 200 eggs for 2–3 days. These offspring are also reared with *ad lib* food until adult and 50 male and 50 females (F1) are selected, mated, and 200 eggs collected. The F2 generation is reared with *ad lib* food to produce F2 adults. F2 adults are selected into individual virgin male: female pairs (family unit). Their offspring (final F3 generation) are then reared at a standardized density (20 eggs) at either high or low food availability until maturation. A wild‐type assay undertaken prior to the experiments start (week 0) underwent a similar methodology but measured offspring from 10 families as opposed to 7. For the purposes of analysis, these data were assigned to each population tube in the experiment to represent an “average” start point for all the populations. Any families with zero female survival in assays were removed from the analysis, such that in some assays there are only data from 5 or 6 families and not 7. As such, the total observations of plasticity are:

n=(1timepoint×10families×3populations×3treatments)+(4timepoints×7families×2populations×3treatments)‐removed(4families)=90+168=258.

Plasticity was measured using reaction norms, obtained from trait values at two ends of an environmental gradient, availability, that is, high and low food availability (Stearns & Koella, 1986; Valladares et al., 2006). We used the slope value of reaction norms to estimate plasticity for size‐at‐maturity and age‐at‐maturity. Common garden assays (outlined above as per Cameron et al., 2013) meant that the slopes are independent assessments of plasticity in trait expression at any given time (i.e., the families measured were not the same at each point in the time series; Figure 1). Therefore, all families are replicate measures of plasticity nested in source populations and nested within treatments.

We also considered variation between family plasticity within a source population as a proxy to ask whether environmental variation selects for, or against, genotypic diversity in a density‐dependent population, as this could also affect how populations respond to novel environments.

### 2.4 Experiment 2: Population responses to novel environments

**FIGURE 3:**
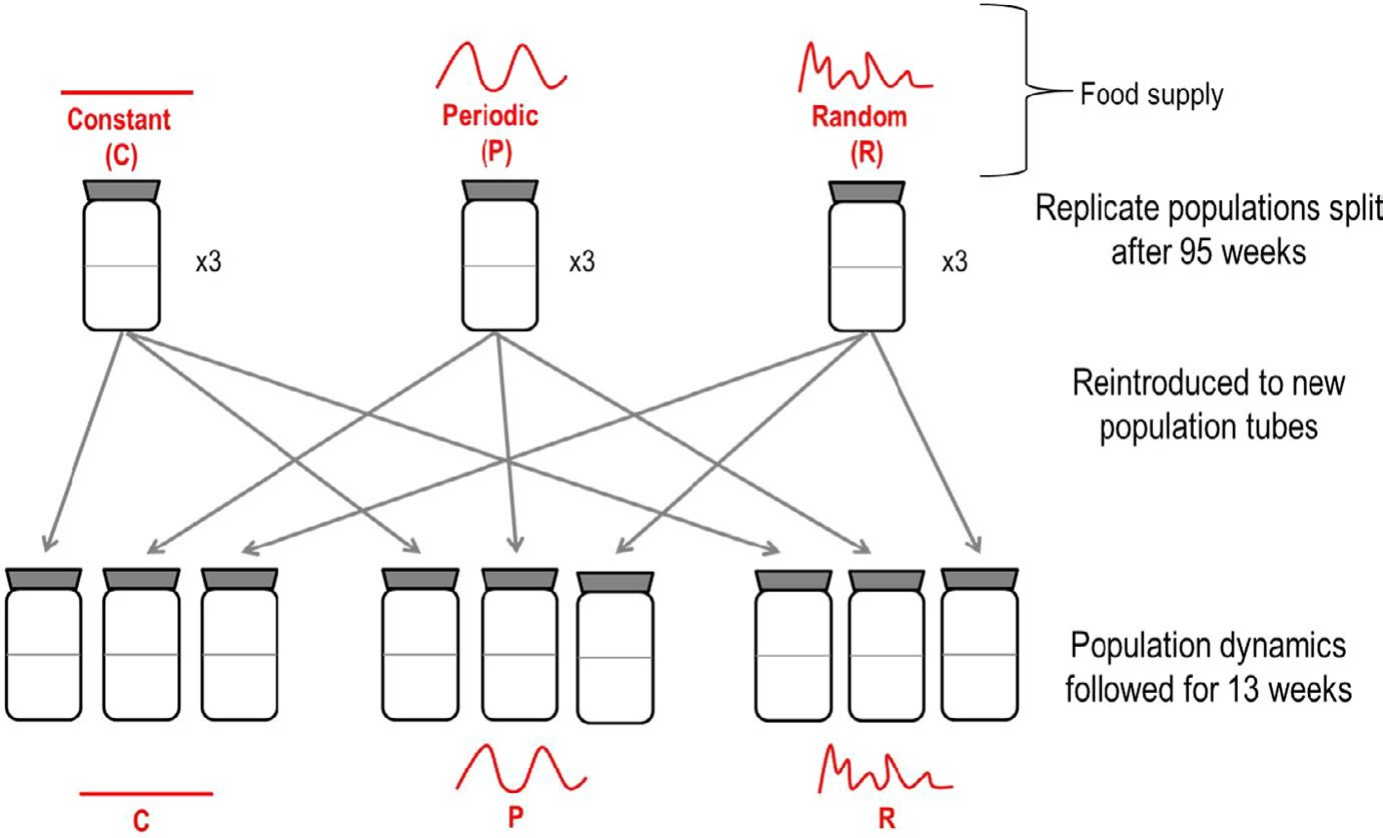
Schematic showing the protocol for Experiment 2—the introduction of experimentally evolved mite populations to novel environments. The mites from three replicate (of the original 6) populations from constant, periodic, or randomly variable environment were each split into new tubes where each one receives either the constant, periodic, or random environment forcing—thus giving rise to 27 timeseries of population abundance

In order to assess the ecological consequences of any selection for plasticity in life history, we inoculated new population tubes with individuals from the original treatment populations at the end of the 95‐week experiment 1. Individuals from these original population selection tubes (three tubes per treatment group, 9 in total) were split equally across three new tubes creating 27 *novel environment* tubes in total. These novel environment tubes were randomly assigned one of the three original environmental treatments and two novel treatments. This created nine treatments, for example, constant–constant, constant–periodic, constant–random, and the same with the other original treatments (Figure 3).

Censuses were conducted weekly for 13 weeks, counting population size of juveniles, adults, and therefore total population size each week for 13 weeks. The coefficient of variation of each stage and total population was calculated from each time series and then averaged across treatment replicates. This experiment tests the role of evolved plasticity in moderating variation in observed population dynamics.

### 2.5 Data analysis

The significance of temporal trends in age and size plasticity and effects of environmental variation on that plasticity were determined using linear mixed‐effects (LME) models using the “nlme” package in R (Pinheiro *et al*, 2019), accounting for repeated measures of life history plasticity nested within population tubes taken as a random effect on the intercept (Cameron et al., 2013). A random slope does not apply here as the families we conduct our life history assays upon are generated uniquely at each point of the time series. By selecting families at random, from population selection tubes following a three‐generation CG rearing, family is our level of replication and each family at each assay point is fully independent of each other. Post hoc comparisons were taken from the summary table of coefficients from each LME, following any required model simplification, with associated Student *t* statistics or comparison of mean differences between treatments.

To assess whether there were differences in the variation of plastic phenotypes between treatments, 95% confidence intervals were generated around the arithmetic mean of the coefficient of variation (CV) between family slopes per treatment, by bootstrap resampling with replacement (*n* = 1,000). Where mean CV is overlapped by 95% confidence intervals from other treatments, we do not consider them to be different.

The relative importance of the original selection environments versus novel tube environments in effecting variation in population dynamics was tested. This was undertaken by using a linear model (ANOVA, CV ~Original*Novel) for total, adult, and juvenile mite variation in abundance. A series of model simplification deletion tests were undertaken to find the minimum adequate model. All analyses and plots were conducted in RStudio (2020).

## 3 RESULTS

### 3.1 Experiment 1: Evolved changes in plasticity

**FIGURE 4:**
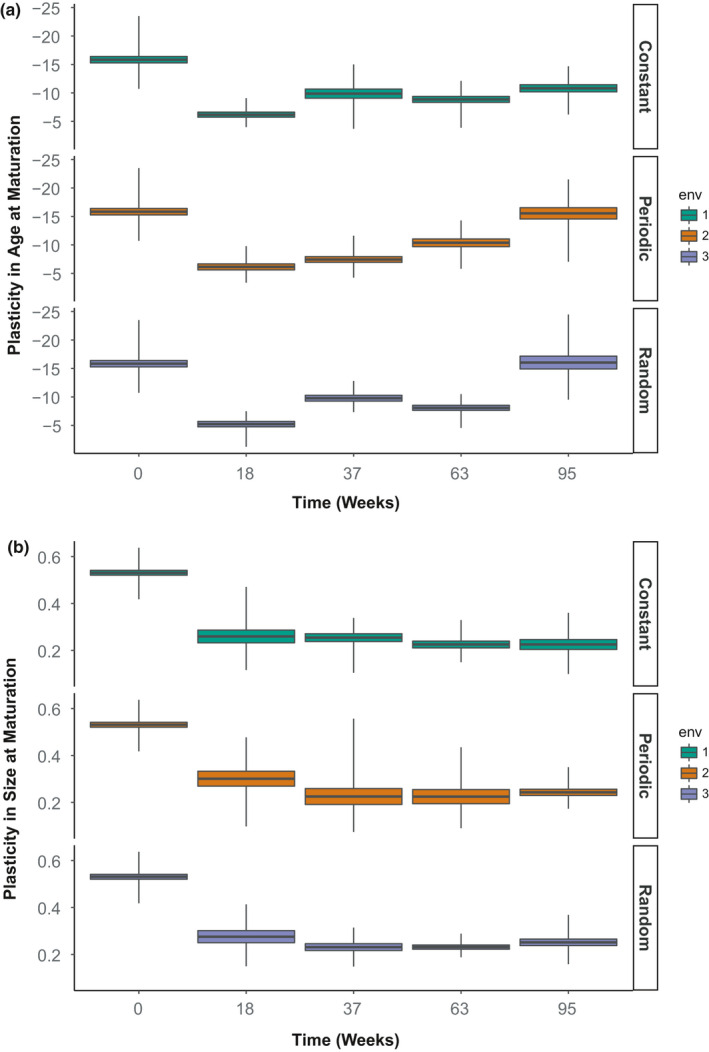
Changing prevalence of plastic genotypes over time, facets showing mean plasticity across families raised in constant, periodic, and random environments. Boxplots show the mean ± 1 standard error, with whiskers indicating max and min. Figure (a) following initial decline shows increasing age plasticity with greatest observed in random and periodic environments which did not differ from one another. Figure (b) shows no recovery for size plasticity (linear mixed‐effects model, 2 populations per treatment per timepoint)

There was a significant change in the life history trait plasticity expressed by F3 offspring throughout the course of the experiment (Figure 4). Initial assessment of plasticity in both age and size showed high levels of plasticity in wild mites just prior to imposing environmental treatments (Age plasticity: −15.84 ± 0.32 (this and all values that follow are mean ± standard error), Size plasticity: 0.53 ± 0.0059). We observed an initial reduction in age and size plasticity, from these initial wild genotypes after a period of 18 weeks across all treatments.

**FIGURE 5:**
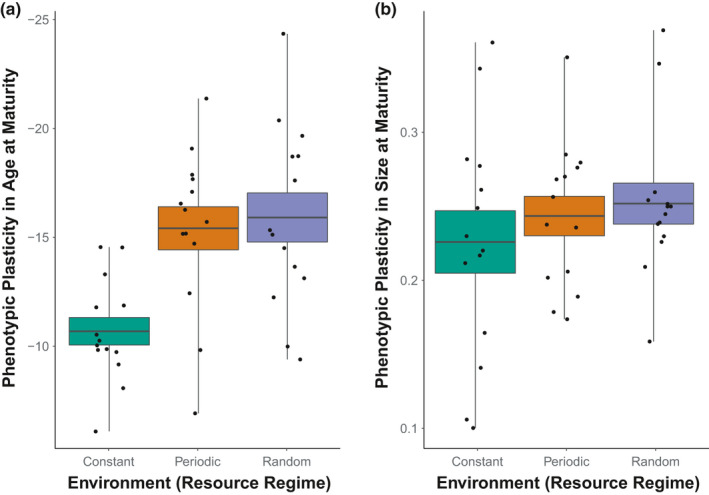
Phenotypic plasticity from common garden rearing in age‐at‐maturity (a) and in size‐at‐maturity (b) at experiment’s end (week 95 in Figure 4). Boxplots show the mean ± 1 standard error, with whiskers indicating max and min. Age plasticity was significantly greater in more variable environments but did not differ between periodic or random environment whereas environmental variation showed no significant effect on size plasticity (linear mixed‐effects, *n* = 7 per population, 2 populations per treatment)

No further change in size plasticity after the 18‐week time point was observed between any of the treatment populations (size plasticity ~assay timepoint * environmental variation: *F*_6, 233_ = 0.15, *p >* .05; Figures 4b and 5b). Age plasticity recovered in all environment treatments over time; however, no significant difference is observed between treatments until the final assay at the end of the experiment in week 95 (lme: age plasticity~assay timepoint * environmental variation: *F*_6, 233_ = 5.187, *p* < .01; Figure 4a). While age plasticity in both random and periodically variable populations was found to be greater by the end of the experiment than in constant environment populations (random environment at end of experiment only—*t*_6, 36_ = −3.93, *p* < .05; periodic—*t*_6, 36_ = −3.56, *p* < .05), they did not differ from each other in their age plasticity (mean difference = 0.5 ± 1.25 *SE*).

### 3.2 Phenotypic diversity—variation in plasticity within populations

**FIGURE 6:**
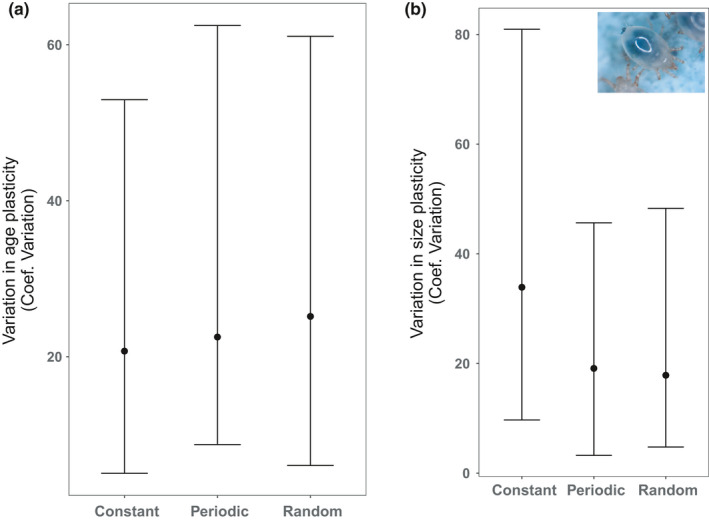
Phenotypic diversity in plasticity for both age (a) and size (b) plasticity at week 95 (experiments end). Figure shows the arithmetic mean coefficient of variation (across *n* = 7 families from each of *n* = 2 treatment populations) with error bars showing 95% confidence intervals after bootstrap resampling with replacement (*n* = 1,000). Overlapping error bars indicate no significant difference in phenotypic diversity

Both random and periodic populations had a higher degree of interfamily variation in age plasticity reaction norm slopes (coefficient of variation: 25.1 and 22.5, respectively) than populations from constant environments (CV: 20.7). Conversely, interfamily variation in size plasticity reaction norm slopes was lowest in populations from variable environments (random CV = 17.9 and periodic CV = 19.1) compared with those from constant environments (CV = 33.9). Bootstrapped resampling estimates of the confidence intervals of these variation estimates do not support these differences being statistically significant (Figure 6). Visualization of reaction norms for families over time shown in Figures 3 and 4 are all available in the supplementary materials (see Figures S2, S3 and S4).

### 3.3 Experiment 2: Population responses to novel environments

Populations of mites that had been raised at one of three levels of original environmental variation (e.g., constant, random, or periodic) were inoculated into new population tubes assigned to one of those same environments. As such, each original population produced three new populations, one in the same environmental condition as before and two novel environments. Differences in population variability were assessed as a function of the original environmental treatment that the mite lines had come from and the novel environment.

**FIGURE 7:**
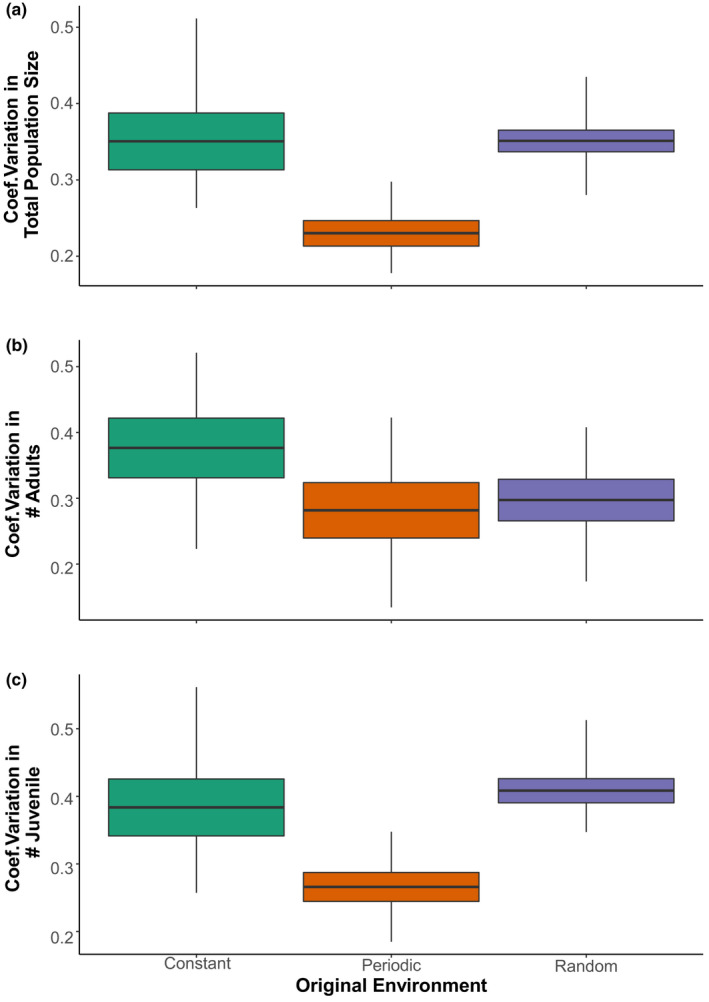
Variation in total population size, adult, and juvenile population size in populations that originated from constant, periodic, and random environments. Boxplots show the mean ± 1 standard error, with whiskers indicating max and min. Plots show variation without control populations, for example, control into control. On average, populations that originated from periodic environments had lowest variation in population sizes (ANOVA, *n* = 6 population tubes per treatment). Treatment time series data are available in the SOMS

The environmental treatment that populations originated from had a significant effect on total population variation (CV ~original environments: *F*_2, 24_ = 5.53, *p* < .01), unlike the novel environment they were introduced to (CV ~Novel Environments: *F*_2, 24_ = 3.22, *p* > .05). Variation in juvenile population abundance was also found to be affected by the original environmental treatment but not the novel environmental treatment (Original: *F*_2, 24_ = 8.42 *p* < .001 vs. Novel: *F*_2, 24_ = 0.85, *p* > .44, Figure 7). For example, populations that originated from periodic environments had 72% less variation in juvenile population size than those from constant, and 70% less variation than random environments. A common pattern was that populations originating from periodic environments tended to have variation that was significantly lower than populations that originated from constant or random environments when exposed to novel tubes (Figure 7). Data displayed here do not include control populations, for example, constant into constant. Please see Figure S3 in the supplementary material for full results that include control populations and Figure S4 for raw time series data for total population size. There was no interactive effect of the original and novel environments on population variability in total abundance (ANOVA, CV ~Original*New, *F*_4, 18_ = 0.54, *p* > .05) or stage abundance (*p* > .05).

## 4 DISCUSSION

We have shown a clear effect of differences in environmental variation leading to different plastic phenotypes evolving in a multigeneration experiment. Through the use of a three‐generation common garden assay environment, then rearing soil mites from birth at either low (High food) or high (Low food) competition—we have demonstrated that higher levels of environmental variation in density‐dependent resource competition selects for greater plasticity in developmental growth rate to maturity. We have also shown that despite no statistically significant differences in the effect of environmental variation on genotypic or family phenotype diversity, there is a trend for populations in the most variable and predictable environment to retain higher interfamily variation in developmental growth (i.e., age plasticity). Those populations that have retained greater plasticity in developmental traits have more stable population dynamics when exposed to novel environments. This is an interesting proof of concept where we have shown an eco‐evolutionary loop (Cameron et al., 2014; Post & Palkovacs, 2014). This loop is simultaneously selecting on components of life history trait plasticity as we have demonstrated here, as well as on mean trait values as has been demonstrated in other studies (e.g., Cameron et al., 2013).

Investigating life history traits and their plasticity is often problematic in wild systems due to logistical constraints and also time required to observe responses. Invertebrate model systems are commonly used to examine population dynamics and selection on trait values due to their short generation time (Beckerman et al., 2010; Robinson & Beckerman, 2013). Soil mites in particular (*S. berlesei*) have long been used as a model organism in ecology and evolution (Benton et al., 2001; Cameron & Benton, 2004). Previous analysis of time series and mean trait values from experimental soil mite populations has shown that evolution of development rate has significant consequences for feedbacks to mean trends in population dynamics, including preventing extinction in novel environments (Cameron et al., 2013). This result was driven by selection for maintaining highest potential fecundity at sexual maturity, by slowing development in highly competitive environments.

Previous studies show that the life history traits of mites when moved from their wild‐type conditions to highly competitive laboratory conditions evolve a delayed age‐at‐maturation. The average trait values expressed in high‐food CG conditions would also have been maladaptive in these wild‐type mites at the start of the experiment, as we found declines in both age and size plasticity as population size declines, and as genetic diversity is lost (Cameron et al., 2013), during the initial stage of the long‐term experiment.

After this initial decline in plasticity in all environments, we see a greater prevalence of families that are highly plastic in their age‐at‐maturation in all subsequent life history assays. The effect of this increase in plasticity is greatest in the most variable environments. Plasticity is costly, with shifts in the mean age‐at‐maturation in low food environments of up to 79% associated with increase in the mean age of high food of up to 69%—increased plasticity is associated with a permanent loss of ability to grow fastest to early maturation.

We found no change in plasticity for size at maturation in any environment. We can place these results in the context of previously reported evolution of the mean trait values of age and size at maturity, where significant evolution of increased age‐at‐maturity (in low food environments) is observed over the course of the experiment, but not in body size (Cameron et al., 2013, 2014). This was driven by density‐dependent competition, where on average all individuals are experiencing food shortage. Delayed growth to maturity was associated with increased fecundity in low food environments—that is, those CG conditions that are more likely to represent the density‐dependent microcosm conditions in which the mites evolved during the experiment (Cameron et al., 2013, 2014). While larger adult mites can have far greater fecundity (Plaistow et al., 2007), this does not apply in low food conditions where body size confers no such advantage (Cameron et al., 2013; Plaistow et al., 2007). More generally, given the mean competitive conditions, investment in body size may be detrimental due to starvation risks as larger individuals have larger metabolic requirements (Bystrom et al., 1998). Assuming that there was sufficient genetic diversity associated with body size, and the decline in body size and plasticity at the bottleneck was adaptive, this perhaps explains why we saw little selection on the mean or plasticity of size‐at‐maturity.

Variable environments have been found to correlate with variable life histories when observed in a natural setting (Hendry, 2016). Aquatic invertebrates exhibit plasticity in life histories in response to variable cues of predation pressure (Beckerman et al., 2010). Indeed, plastic responses in maturation and growth have been observed in environments that are characterized by their variability, such as in rainfall events (Furness et al., 2015) and in thermal regimes (Hoving et al., 2013). However, direct empirical evidence demonstrating that the variability of environments is selecting for flexibility in life history strategies is lacking (Hendry, 2016). In this study, we observed pronounced effects of environmental variation on the evolution of plasticity in age‐at‐maturation. In this instance, flexibility in growth rate allows an individual to capitalize on resources when they are high but also facilitates persistence when resources are low. These observations have been observed in comparative studies of aquatic invertebrate (Zhang, 2006) and fish populations (Gale et al., 2013) in response to altered resource availability suggesting that our results are more generalized and that environmental variation may maintain plasticity in a variety of taxa.

We did not see any difference in the evolution of plasticity values between the random and periodic environments, that is, the stochastic and predictable variable environments. Stochastic environments that are unpredictable in nature are said to favor bet‐hedging strategies as opposed to plasticity in development (Furness et al., 2015). Diversified bet hedging allows a female to produce offspring that can express a range of specific phenotypes, that is, many offspring, each expressing phenotypes optimal for a particular environment so that at least a portion of offspring survive (Einum & Fleming, 2004). However, given our methodology we may not have been able to detect bet‐hedging. It is difficult to differentiate between the two strategies, as the allocation of female eggs to either high or low food life history assay conditions was entirely randomized, but a shift in average assay trait measurements was expected.

Evolved changes in mean values of maturation life history traits are well documented to have feedbacks on population dynamics that may promote persistence or productivity of systems (Cameron et al., 2013; Quetglas et al., 2016; Reznick et al., 2001). Given that plasticity may also be selected for if it improves fitness in variable environments, the role of plasticity in life history traits is increasingly relevant in examining eco‐evolutionary dynamics (Richter et al., 2012; Torres‐Dowdal et al., 2012). In rotifer–algae predator–prey systems, predator‐induced plastic responses in prey defense and growth rate were found to feedback on predator–prey cycles (Fischer et al., 2014). What is less clear is whether selection for plasticity, or increases in the frequency of developmentally plastic genotypes in a population, will either reduce or enhance the variability of population dynamics in a given population. Plasticity could be considered adaptive if it was to reduce the likelihood of excessively poor conditions an individual or their offspring was to experience—as would occur if population variability were to decline. By reintroducing mites from given background environments to novel environments, we have shown the role that plasticity can play in eco‐evolutionary dynamics. Variation in population sizes, total population and specific life history stages, indicated the original environment that mite populations experienced most influenced the ability of individuals to respond to novel environments and therefore the dynamics of those populations. Mite populations originating from periodic environments showed lowest overall variation in total population size when moved to a novel environment. This suggests that either the tendency for higher plasticity found in those populations, or some unmeasured dimension of the plasticity, had greatest capacity to reduce the environmental variation experienced by individuals from periodic environments when colonizing new environments. This was likely driven by a similar result (low variation in periodic mites) in juvenile counts, indicating that the ability to delay growth to maturity also led to reduced variation in juvenile abundance. This will have also been contributed to by a feedback from adult reproduction rates—the more juveniles alive, the more severe competition for food, and the lower adult fecundity becomes.

Variation in adult population sizes remained high in novel environments regardless of the environment that mites had originated. This can be explained by the high age plasticity we observed in our final life history assay. The aforementioned age plasticity combined with the food regime would result in high variation in the number of recruits into the adult stage. As such, our results support previous work highlighting the potential importance of plasticity in maturation rates for the persistence of populations—through reducing the potential negative effect of environmental variation on individual fitness (Aratayev & Raft, 2015). Intriguingly while overall plasticity in age‐at‐maturation evolved to similar levels in both the periodic and random environments, this did not transfer into similar results in population variation when mites were moved to novel environments. Mites from random environmental backgrounds did not experience the same range of positive effects of reduced variation in abundance in novel environments than those originating from periodic environments. This points to all plasticity *not being equal*, and further research on this lack of equality is warranted.

These results shed light on the role of environmental variation in maintaining plasticity even when strong directional selection is operating. Environments are never entirely constant, random or periodic—even constant environments can result in an experience of environmental variation due to demographic stochasticity (Cameron et al., 2014). Given our observations here, we conclude that plasticity may reduce the likelihood of environment‐induced extreme population densities.

## 5 CONCLUSION

We have used an established invertebrate model system to empirically confirm environmental variation in density‐dependent resource use can result in evolved changes in phenotypic plasticity in two key life history traits, age, and size at maturation. Additionally, we have shown how evolved plasticity affects the response of a population to a novel environment and how the response of population dynamics helped explain the maintenance of phenotypic plasticity—even in the presence of strong directional selection. Combined, these two main results evidence the importance of considering selection on phenotypic plasticity when predicting the eco‐evolutionary dynamics of populations in a changing world.

## CONFLICT OF INTEREST

None declared.

## AUTHOR CONTRIBUTION

**Matthew N Bond:** Formal analysis (lead); Writing‐original draft (lead); Writing‐review & editing (lead). **Tom C. Cameron:** Methodology (equal); Project administration (equal); Supervision (lead); Writing‐review & editing (equal). **Tim Benton:** Conceptualization (equal); Funding acquisition (lead); Methodology (equal); Project administration (equal); Supervision (supporting); Writing‐review & editing (equal). **Stuart B. Piertney:** Conceptualization (equal); Funding acquisition (equal); Investigation (equal); Methodology (equal); Project administration (equal); Supervision (supporting); Writing‐review & editing (equal).

## Supporting information

Supplementary Material

## Data Availability

All data used in this study are made available through the publicly available web portal—the University of Essex Research Data repository(http://researchdata.essex.ac.uk/).
