# Supplementary material for: Plasticity is a locally adapted trait with consequences for ecological dynamics in novel environments
Source: Ecol Evol. 2021 Jul 21;11(16):10868–79. doi: 10.1002/ece3.7813 (PMC8366859; doi:10.1002/ece3.7813)
Supplement: Supplementary file 1 — Supplementary Material [file ECE3-11-10868-s001.docx]

**Supporting information for “Plasticity is a locally adapted trait with consequences for ecological dynamics in novel environments”**

**Introduction**

This supplementary information (SI) is supplied to give additional information about our study system, methods, and analyses for the manuscript titled “Plasticity is a locally adapted trait with consequences for ecological dynamics in novel environments”.

1. **Methods**

**Model system**

Here we use the soil mite (Sancassania berlesei) model system to investigate the role of environmental variation on the evolution of phenotypic plasticity in life history traits. Soil mites in microcosms are known to exhibit high levels of intraspecific competition, with adults and juveniles in direct competition for resources (Benton 2012). Juveniles are distinct from adults by relative body size and different shaped appendages (Plaistow et al. 2004). Adults are competitively dominant over juveniles, as increased body size allows for dominance of food patches. Therefore, body size is a highly canalized trait within a competitive microcosm population. However, in previous studies (Plaistow et al. 2006), soil mites show evidence of plastic life history traits associated with growth in response to resource allocation. This model system lends itself well to the study of evolution in contemporary time due to short life cycles (10-50 days), and small space required to house replicate populations(Cameron & Benton 2004). General laboratory and husbandry techniques associated with this species can be found in previously published work (Benton et al. 2001).

In this study we have developed estimates of plasticity in maturation traits from a data set previously analysed for a different set of questions. While temporal dynamics of the life history traits has been considered – this is the first time the plasticity in these same traits have been estimated and analysed in the context of environmental variation. We then report on an entirely new and unpublished experiment where mites from populations held in different conditions for over ~13-30 generations are then inoculated into new population tubes and manipulated to be either their same or novel environments.

**Quantification of Phenotypic Plasticity**

Figure S1 shows an example of how plasticity in Age and Size at Maturation was quantified. Trait values were generated through the use of a three-generation common garden rearing experiment, detailed in Cameron et al (2013). Trait values were collected at high and low food from the offspring of a given family. We then used a linear model to calculate the slope value and therefore a plasticity value for each family that arose from multiple common gardens conducted during the course of Experiment 1 outlined in the main manuscript.


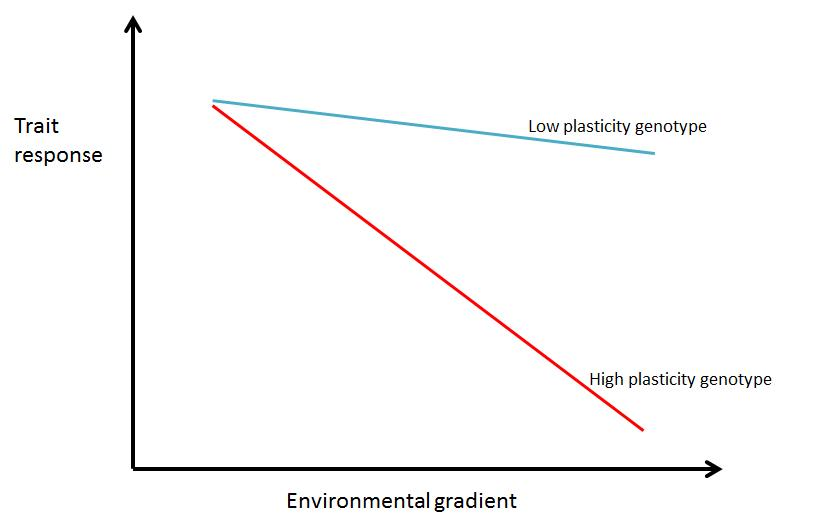


Figure S1: Diagram to illustrate reaction norms as a way to quantify plasticity. The steeper slope indicates greater plasticity and therefore has a greater slope value.

**3. How plasticity at the treatment level was compared**

In the main manuscript we discuss “phenotypic variation”, i.e. variability between families in how plastic they are for a given trait. Methods in the main manuscript describe how this was calculated with bootstrap resampling.

To illustrate this, we also present here the raw phenotype data over time shown by individuals reared in both high and low food environments at the end of each 3 generation common garden (Figures S2 and S3). In addition, we display the reaction norms for each family in the final assay for Experiment 1 (Figure S4).


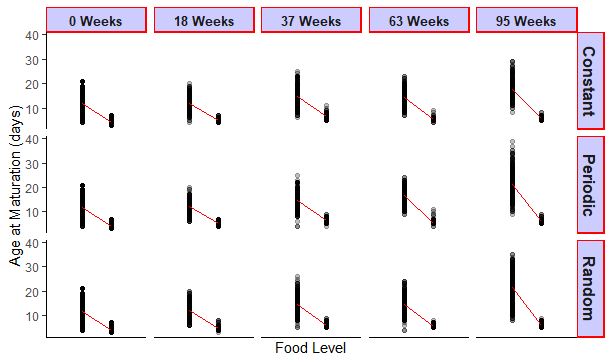
Figure S2: Age at maturation for individuals reared in high and low food environments at the end of a 3-generation common garden rearing experiment. The red line indicates the mean reaction norm for each environmental treatment at each assay time point.


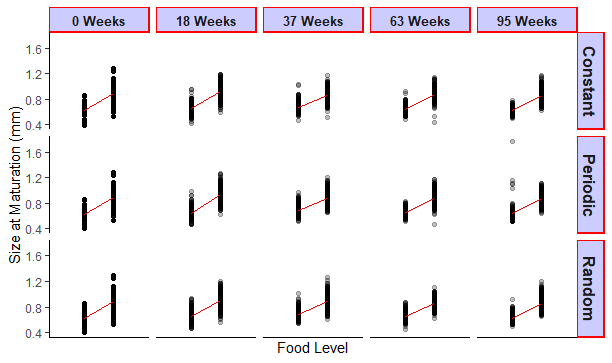


Figure S3: Size at maturation for individuals reared in high and low food environments at the end of a 3-generation common garden rearing experiment. The red line indicates the mean reaction norm for each environmental treatment at each assay time point.

Figure S4: Raw reaction norms from common garden rearing experiments at week 95. Families with offspring reared in high and low food are joined with a line to represent the reaction norm for each. Left and right panels indicate reaction norms for Age and Size at maturation respectively.

1. **Additional plot of population variance in novel environments**

Figure S shows the variation in population size in mite populations that have been introduced to novel environments, from their original environmental treatments, both including control populations (e.g. constant into constant), and without controls.


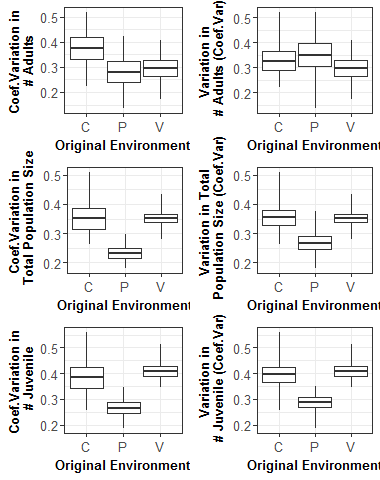


Figure S5: Coefficient of Variation in adult population size (top row), total population size (middle row) and juvenile population size (bottom row). Left panel includes control populations (e.g. periodic populations put into periodic environments) and the right does not include control populations.

1. **Additional plot of raw time series data for total population size in novel environments**

Figure S6: Time series of total population size for reintroduced populations originating from either Constant (left), Periodic (middle) or Randomly (right) variable environments.
